# Central Glucagon-like Peptide-1 Receptor Signaling via Brainstem Catecholamine Neurons Counteracts Hypertension in Spontaneously Hypertensive Rats

**DOI:** 10.1038/s41598-019-49364-x

**Published:** 2019-09-19

**Authors:** Kenichi Katsurada, Masanori Nakata, Toshinobu Saito, Boyang Zhang, Yuko Maejima, Shyam S. Nandi, Neeru M. Sharma, Kaushik P. Patel, Kazuomi Kario, Toshihiko Yada

**Affiliations:** 1 Division of Integrative Physiology, Department of Physiology, Jichi Medical University School of Medicine, Shimotsuke, Tochigi 329-0498 Japan; 2 Division of Cardiovascular Medicine, Department of Internal Medicine, Jichi Medical University School of Medicine, Shimotsuke, Tochigi 329-0498 Japan; 3 Department of Physiology, Wakayama Medical University School of Medicine, Wakayama, 641-8509 Japan; 4 Department of Pharmacology, Fukushima Medical University School of Medicine, Fukushima, 960-1295 Japan; 5 Department of Cellular and Integrative Physiology, University of Nebraska Medical Center, Omaha, NE 68198-5850 USA; 6 Center for Integrative Physiology, Kansai Electric Power Medical Research Institute, 1-5-6 Minatojimaminamimachi, Chuou-ku, Kobe, 650-0047 Japan; 7 Division of System Neuroscience, Kobe University Graduate School of Medicine, 7-5-2 Kusunoki-cho, Chuou-ku, Kobe, 650-0017 Japan

**Keywords:** Neuroscience, Metabolic syndrome

## Abstract

Glucagon-like peptide-1 receptor (GLP-1R) agonists, widely used to treat type 2 diabetes, reduce blood pressure (BP) in hypertensive patients. Whether this action involves central mechanisms is unknown. We here report that repeated lateral ventricular (LV) injection of GLP-1R agonist, liraglutide, once daily for 15 days counteracted the development of hypertension in spontaneously hypertensive rats (SHR). In parallel, it suppressed urinary norepinephrine excretion, and induced c-Fos expressions in the area postrema (AP) and nucleus tractus solitarius (NTS) of brainstem including the NTS neurons immunoreactive to dopamine beta-hydroxylase (DBH). Acute administration of liraglutide into fourth ventricle, the area with easy access to the AP and NTS, transiently decreased BP in SHR and this effect was attenuated after lesion of NTS DBH neurons with anti-DBH conjugated to saporin (anti-DBH-SAP). In anti-DBH-SAP injected SHR, the antihypertensive effect of repeated LV injection of liraglutide for 14 days was also attenuated. These findings demonstrate that the central GLP-1R signaling via NTS DBH neurons counteracts the development of hypertension in SHR, accompanied by attenuated sympathetic nerve activity.

## Introduction

Glucagon-like peptide-1 (GLP-1) related medicines are widely used to treat type-2 diabetic patients. Recently, cardiovascular outcome study of GLP-1 receptor (GLP-1R) agonists showed reduction of cardiovascular events and prevention of chronic kidney disease progression in type-2 diabetic patients^1–3^. Studies in hypertensive patients with type-2 diabetes showed that long-term treatment with GLP-1R agonists reduced blood pressure (BP)^4–10^. This antihypertensive effect of GLP-1R agonists might contribute to the ability of these medicines to protect the cardiovascular system.

Several possible mechanisms for antihypertensive effect of GLP-1R agonists have been postulated. GLP-1R agonist directly acts on the kidney to induce natriuresis^11–14^, on the endothelial cells to induce vasodilatation^12,15^, and on the atrial cardiomyocytes to induce secretion of atrial natriuretic peptide^16^. In addition to these direct effects on the end effectors, GLP-1 may also regulate BP via the central action. GLP-1Rs are expressed in the hypothalamus and brainstem regions implicated in sympathetic and cardiovascular control^17,18^. GLP-1R agonists can pass the blood brain barrier^19–21^. However, the mechanisms underlying the central action of GLP-1R agonists to lower BP remain to be fully elucidated.

To date there have been two clinical trials showing suppression of cardiovascular events in type 2 diabetic patients treated with GLP-1R agonists, LEADER^3^ and SUSTAIN-6^2^ trials. In LEADER trial, GLP-1R agonist, liraglutide significantly decreases systolic BP (SBP) by −1.2 mmHg and suppresses death from cardiovascular causes. In SUSTAIN-6 trial, another GLP-1R agonist, semaglutide that has same amino-acid sequences as liraglutide significantly decreases SBP by −3.4 to −5.4 mmHg and suppresses nonfatal stroke. It is well known that development and progression of cardiovascular disease is strongly associated with sympathetic hyperactivity and treatment with sympathoinhibition probably leads to suppression of cardiovascular events^22,23^. Further, these strong and definite cardiovascular effects of liraglutide could be independent on the glucose lowering and associated effects. Based on these results form LEADER and SUSTAIN-6 trials we hypothesized that liraglutide exhibits antihypertensive effect accompanied with sympathoinhibition leading to suppression of cardiovascular events.

To test this hypothesis we assessed the central action of liraglutide and underlying mechanism to counteract hypertension in spontaneously hypertensive rat (SHR) that has hypertensive and sympathoexcitative properties. We performed intracerebroventricular (icv) injection of liraglutide (0.9 µg/3 µl) once daily for 15 days, and investigated its effect on urinary norepinephrine excretion, as a marker of systemic sympathetic nerve activity, and on c-Fos expression in the hypothalamus and brainstem of central nervous system (CNS) as a marker for neuronal activity in the CNS. To gain further insight into the central neuron mediating the GLP-1 action, we focused on the nucleus tractus solitarius (NTS) catecholamine neurons expressing dopamine beta-hydroxylase (DBH), since DBH converts dopamine to norepinephrine and the NTS DBH neuron is implicated in regulation of cardiovascular functions. It has been reported that reduced DBH activity in the brain is linked to an increase in blood pressure^24–27^, and that lesion of NTS DBH neurons induces baroreflex dysfunction and liability for BP^28,29^. To delete NTS catecholamine neurons, we injected the saporin-conjugate antibody to DBH (anti-DBH-SAP) into NTS bilaterally and investigated whether the effect of icv liraglutide on BP is affected.

We found that repeated lateral ventricular (LV) injection of liraglutide counteracted the development of hypertension and suppressed sympathetic nerve activity. Fourth ventricular (4V) injection of liraglutide acutely decreased BP. The lesion of NTS DBH neurons by anti-DBH-SAP attenuated both acute depressor effect of 4V injection of liraglutide and chronic antihypertensive effect of LV injection of liraglutide.

## Results

### Repeated LV injection of liraglutide attenuates development of hypertension in SHR

**Figure 1:**
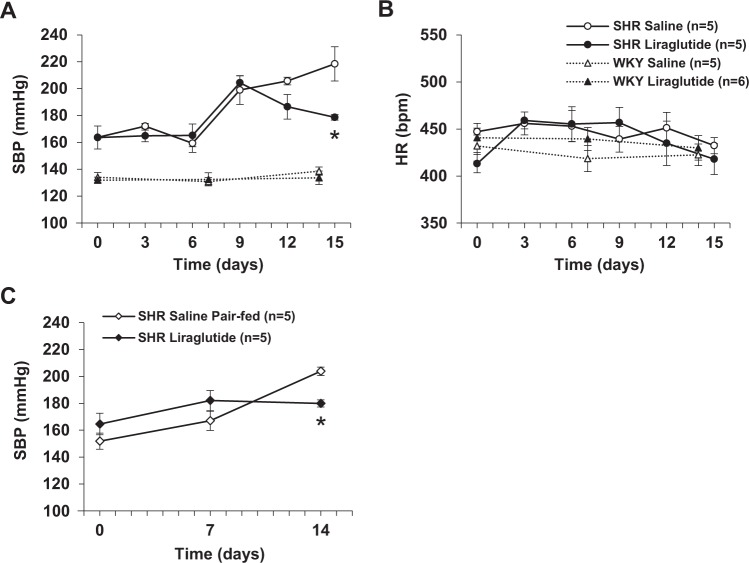
Repeated LV injection of liraglutide attenuates development of hypertension in SHR. Effect of repeated LV injection of 0.9 µg liraglutide or saline once a day for 15 days on systolic blood pressure (**A**) and heart rate (**B**) in SHR (n = 5) and WKY rats (n = 5–6), and systolic blood pressure (**C**) in liraglutide-injected SHR (n = 5) and saline-injected SHR pair-fed to liraglutide-injected group (n = 5). Data are shown as mean ± SE. *P < 0.05 between liraglutide and saline groups by repeated measures two-way ANOVA followed by Turkey’s post hoc test. Figure A (SHR): P value for time, group and time x group interaction are p < 0.0001, p = 0.0409 and p = 0.0301, respectively. Figure C: P value for time, group and time x group interaction are p < 0.0001, p = 0.7918 and p = 0.0045, respectively.

SBP in SHR significantly increased during the 15 days period. Repeated LV injection of liraglutide (0.9 μg/3 µl) once a day attenuated the increase of SBP in SHR at 15 days of injection, and had no effect on SBP in control Wister Kyoto (WKY) rats (Fig. 1A). Administration of liraglutide did not influence heart rate (HR) in both SHR and WKY rats during the 15 days period (Fig. 1B). In the pair feeding study, the pair-fed saline injected SHR exhibited significant increase in SBP during the 14 days period, similar to the saline injected SHR without pair feeding (Fig. 1C). Repeated LV injection of liraglutide counteracted the increase in SBP in SHR at 14 days of injection (Fig. 1C).

### Repeated LV liraglutide suppresses urinary norepinephrine excretion in SHR

**Figure 2:**
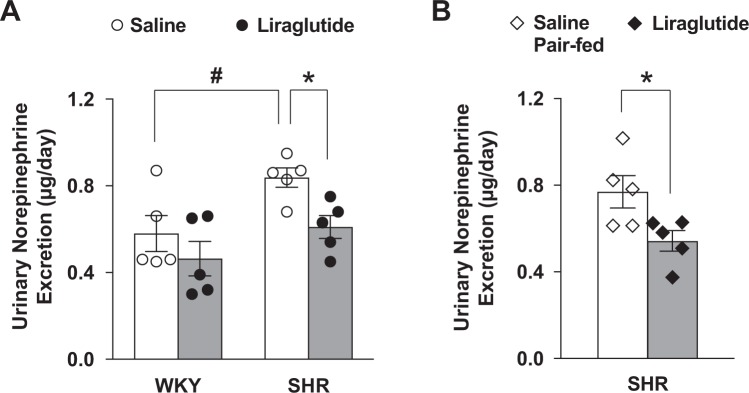
Repeated LV liraglutide suppresses urinary norepinephrine excretion in SHR. Effect of repeated LV injection of liraglutide for 14 days on the 24 hour urinary norepinephrine excretion in SHR and WKY rats (**A**, n = 5), and in liraglutide-injected SHR and saline-injected SHR pair-fed to liraglutide-injected rats (**B**, n = 5). *P < 0.05 between liraglutide and saline. ^#^*P* < 0.05 between WKY with saline and SHR with saline.

The urinary norepinephrine excretion was measured at 14 days of repeated LV injection of liraglutide or saline. The urinary norepinephrine excretion was significantly elevated in SHR compared to WKY rats, and this elevation was significantly attenuated by liraglutide administration (Fig. 2A). In the pair feeding study, the pair-fed saline injected SHR exhibited significant increase in norepinephrine excretion (Fig. 2B) to a level similar to that in the saline injected SHR without pair feeding (Fig. 2A). Repeated LV injection of liraglutide significantly suppressed the norepinephrine excretion in SHR compared to pair-fed saline injected SHR at 14 days of injection (Fig. 2B).

### Repeated LV liraglutide induces c-Fos expression in AP and NTS

**Figure 3:**
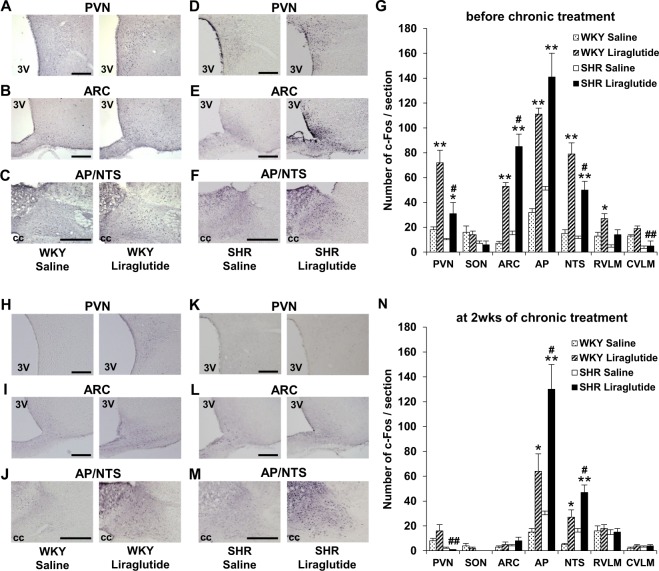
Repeated LV liraglutide induces c-Fos expression in AP and NTS. Distribution of c-Fos expression in the brain of SHR and WKY rats at four hours after LV injection of liraglutide on the first day of experiment (**A–G**, n = 5) and on the next day after termination of repeated LV injection of liraglutide for two weeks (**H–N**, n = 6–7). (**A–F,H–M**) c-Fos immunostaining in PVN, ARC and AP/NTS after LV injection of saline (left panel) or 0.9 µg liraglutide (right panel). Scale bars indicate 200 µm. 3V, third ventricle; CC, central canal. (**G,N**) Number of c-Fos immunoreactive (IR) neurons/section in PVN, SON, ARC, AP, NTS, RVLM and CVLM after injection of 0.9 µg liraglutide or saline. Each bar represents mean ± SE. *P < 0.05, **P < 0.01 between liraglutide and saline, ^#^*P* < 0.05, ^##^*P* < 0.01 between SHR with liraglutide and WKY with liraglutide.

To explore the brain regions targeted by liraglutide, c-Fos expression in the brain was investigated at four hours after LV injection of liraglutide on the first day of experiment. LV injection of liraglutide, compared to saline, significantly increased c-Fos expression in the paraventricular nucleus (PVN) and arcuate nucleus (ARC) in hypothalamus and area postrema (AP) and NTS in brainstem in both SHR and WKY rats (Fig. 3A–G). Later, on one day after termination of repeated LV injection of liraglutide for 14 days, LV injection of liraglutide significantly increased c-Fos expression in the AP and NTS in brainstem, while that in hypothalamic PVN and ARC was unaltered in both SHR and WKY rats (Fig. 3H–M). The number of c-Fos positive cells in the AP and NTS were around 2-fold more in SHR than in WKY rats at 14 days of treatment with liraglutide (Fig. 3N). Thus, LV injection of liraglutide induced antihypertensive effect and c-Fos expression in the AP and NTS at 15 days in SHR.

### Repeated intraperitoneal injection of liraglutide induces c-Fos expression in AP and NTS

**Figure 4:**
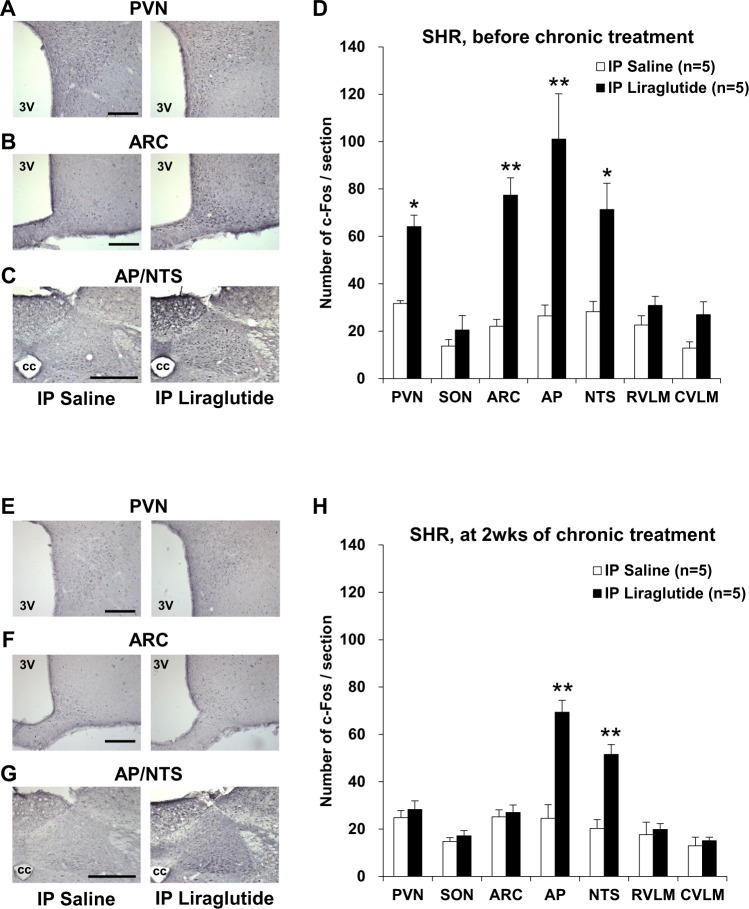
Repeated IP liraglutide induces c-Fos expression in AP and NTS. Distribution of c-Fos expression in the brain of SHR at four hours after intraperitoneal (IP) injection of liraglutide on the first day of experiment (**A–D**, n = 5) and on the next day after termination of repeated IP injection of liraglutide for two weeks (**E–H**, n = 5). (**A–C,E–G**) c-Fos immunostaining in PVN, ARC and AP/NTS after IP injection of saline (left panel) or 90 μg/kg liraglutide (right panel). Scale bars indicate 200 µm. 3V, third ventricle; CC, central canal. (**D,H**) Number of c-Fos-IR neurons/section in PVN, SON, ARC, AP, NTS, RVLM and CVLM after injection of liraglutide or saline. Each bar represents mean ± SE. *P < 0.05, **P < 0.01 between liraglutide and saline.

To assess the area in the brain targeted by peripheral injection of liraglutide, as well as LV injection of it, c-Fos expression in the brain was investigated at four hours after intraperitoneal (IP) injection of liraglutide both before and after repeated IP injection of liraglutide once a day for 14 days. The first IP injection of liraglutide increased c-Fos expression in PVN, ARC, AP and NTS in SHR (Fig. 4A–D). After treatment with liraglutide by repeated IP injection for 14 days, the next IP injection of liraglutide increased c-Fos expression in AP and NTS, but not PVN and ARC (Fig. 4E–H).

### Acute 4V injection of liraglutide decreases blood pressure in SHR

**Figure 5:**
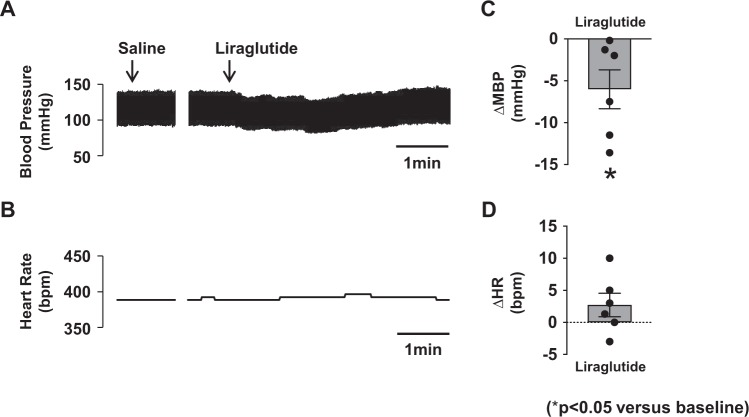
Acute 4V injection of liraglutide decreases blood pressure in SHR. Original recordings of the effect of 4V injection of liraglutide (0.9 µg/3 µl) on blood pressure and heart rate (**A**,**B**) and peak changes in mean blood pressure (MBP) and heart rate (HR) (**C**,**D**) (n = 6). *P < 0.05 vs. baseline.

These results prompted us to hypothesize that liraglutide acts on the AP and/or NTS to counteract hypertension in SHR. Hence, we performed acute administration of liraglutide into 4V, the area with easy access to the AP and NTS, and it decreased the blood pressure measured intra-arterially in anesthetized SHR (Fig. 5A). The change took transient pattern, and the peak change in average of mean arterial pressure (MAP) was −6.0 ± 2.1 mmHg (Fig. 5C). In contrast, 4V injection of liraglutide had no effect on HR (Fig. 5B,D).

### Repeated LV liraglutide activates NTS DBH neurons

**Figure 6:**
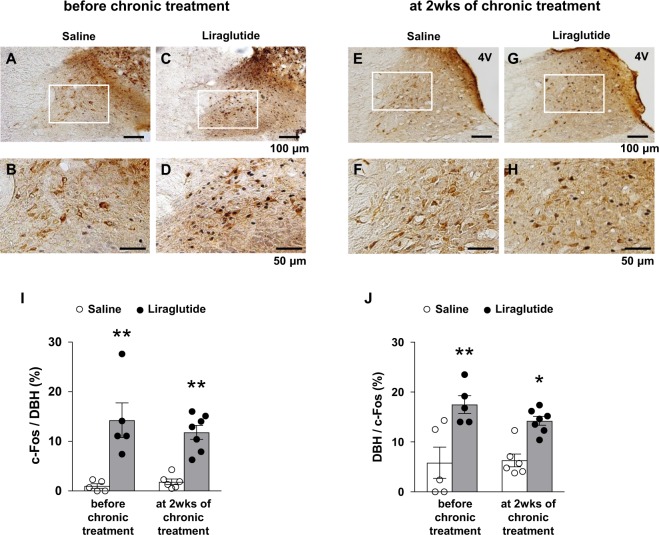
Repeated LV liraglutide activates NTS DBH neurons. LV injection of liraglutide induces c-Fos expression in NTS DBH neurons before and after chronic treatment with repeated LV liraglutide for 2 weeks (wks). Representative images of double immunostaining for c-Fos and DBH in NTS after LV injection of saline (**A,B,E,F**) or liraglutide (**C,D,G,H**). (**B,D,F,H**) Magnified images of the areas specified by white squares in (**A,C,E,G**). Scale bars indicate 100 µm in (**A,C,E,G**), and 50 µm in (**B,D,F,H**). (**I**) Percentage of c-Fos-IR neurons in DBH neurons in NTS after LV injection of liraglutide (filled bar: n = 5–7) or saline (open bar: n = 5–6) before and at 2 wks of chronic treatment. (**J**) Percentage of DBH neurons in c-Fos-IR neurons in NTS after LV injection of liraglutide (filled bar: n = 5–7) or saline (open bar: n = 5–6). Each bar represents mean ± SE. *P < 0.05, **P < 0.01 between liraglutide and saline.

The NTS is one of key centers for cardiovascular control, where DBH neurons play important roles in homeostatic regulation of BP including baroreflex. We focused on the relationship between BP control and NTS DBH neurons under LV injection of liraglutide. We performed double immunostaining for c-Fos and DBH in the NTS in SHR after LV injection of liraglutide on both the first day and one day after termination of repeated LV injection of liraglutide for 14 days. LV injection of liraglutide, compared to saline, significantly increased c-Fos expression in NTS DBH neurons both before and after repeated LV injection of liraglutide (Fig. 6). After repeated LV injection of liraglutide, 12.9% of DBH neurons were c-Fos-immoreacitive (IR) in liraglutide group compared with 1.8% in control group (p < 0.05, Fig. 6I). The fraction of DBH-IR neurons among the c-Fos-IR neurons was significantly increased by liraglutide administration (14.2% in liraglutide group vs. 6.3% in control group, p < 0.05) (Fig. 6J). These results show that LV injection of liraglutide specifically and substantially activates NTS DBH neurons.

### Lesion of NTS DBH neurons attenuates both acute depressor effect of 4V liraglutide and chronic antihypertensive effect of repeated LV liraglutide

**Figure 7:**
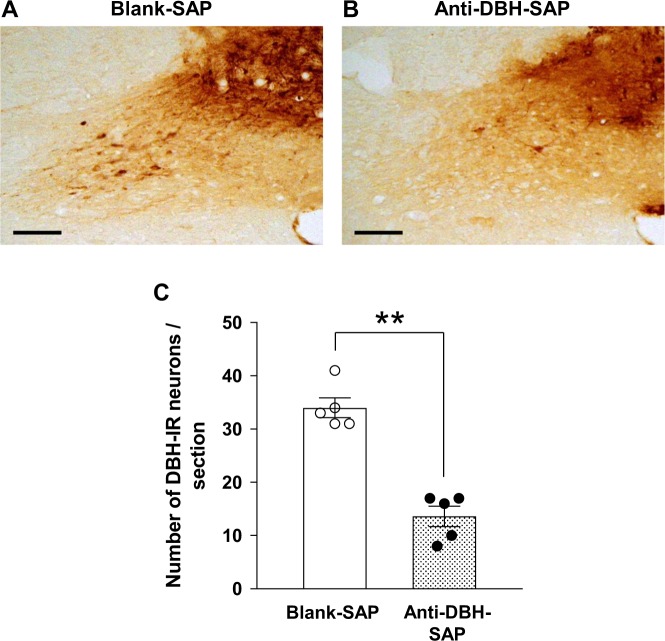
Microinjection of anti-DBH-SAP deletes NTS DBH neurons. Representative images of the DBH immunostaining in NTS after microinjection of blank-SAP (**A**) or anti-DBH-SAP (**B**) into NTS. Scale bars indicate 100 µm. (**C**) Number of DBH-IR neurons/section in NTS was decreased significantly by microinjection of anti-DBH-SAP (n = 5). Each bar represents mean ± SE. **P < 0.01 between anti-DBH-SAP and blank-SAP.

**Figure 8:**
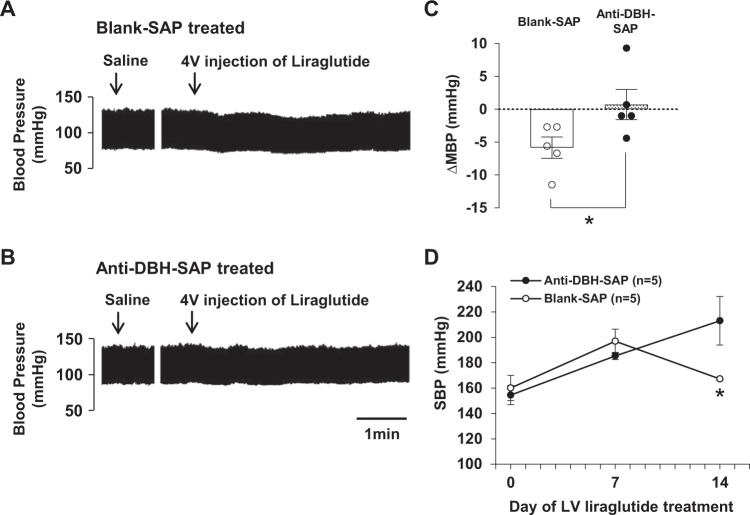
Lesion of NTS DBH neurons attenuates acute depressor effect of 4V liraglutide and chronic depressor effect of LV liraglutide. Original recordings of the effect of 4V injection of liraglutide on blood pressure in SHR treated with blank-SAP (**A**) or anti-DBH-SAP (**B**). (**C**) The peak depressor change in average of MBP after 4V injection of liraglutide was significantly attenuated in SHR treated with anti-DBH-SAP (n = 5). (**D**) The antihypertensive effect of repeated LV liraglutide at 2 wks was significantly attenuated in SHR treated with anti-DBH-SAP (n = 5). Each bar represents mean ± SE. *P < 0.05 between anti-DBH-SAP and blank-SAP by unpaired Student’s t test (**C**) and repeated measures two-way ANOVA followed by Turkey’s post hoc test (**D**). Figure D: P value for time, group and time x group interaction are p = 0.0003, p = 0.2989 and p = 0.0073, respectively.

The DBH-IR neurons were significantly reduced by microinjection of anti-DBH-SAP, compared to control blank-SAP, into the NTS (14 ± 2 in anti-DBH SAP group vs. 34 ± 2 in blank-SAP group, p < 0.01) (Fig. 7A–C). The depressor effect of 4V injection of liraglutide was attenuated in anti-DBH-SAP injected SHR, as compared to that in control blank-SAP injected SHR (∆MAP 0.7 ± 2.1 mmHg in anti-DBH-SAP injected group vs. −5.8 ± 1.4 mmHg in control group) (Fig. 8A–C). The antihypertensive effect of repeated LV injection of liraglutide during 14 days was also attenuated in anti-DBH-SAP injected group compared to control group (Fig. 8D).

## Discussion

We have shown that repeated LV injection of liraglutide once a day for 15 days counteracted the development of hypertension in SHR, suppressed the norepinephrine excretion, and induced c-Fos expressions in NTS DBH neurons. Fourth ventricular injection of liraglutide transiently decreased BP in anesthetized SHR and this effect was attenuated after lesion of NTS DBH neurons by treating with anti-DBH-SAP. In this anti-DBH-SAP injected SHR, the antihypertensive effect of repeated LV liraglutide was also attenuated. This study indicates that sustained GLP-1R activation in CNS decreases BP via activation of NTS DBH neurons and suppression of sympathetic nerve activity in SHR.

The first LV injection of liraglutide increased c-Fos expression in the PVN and ARC in hypothalamus and the AP and NTS in brainstem. This result is in agreement with previous reports^30,31^. In contrast, following repeated LV injection of liraglutide for 14 days, subsequent LV injection of liraglutide enhanced c-Fos expression in the AP and NTS but failed to do so in the PVN and ARC. It is noted that c-Fos expression in AP and NTS was increased in SHR to a 2-fold greater extent than in WKY rats after repeated LV injection of liraglutide for 14 days. Thus, induction of c-Fos in AP and NTS coincided with a suppression of norepinephrine excretion and counteraction of hypertension in SHR. These findings suggest that the greater activation of AP and NTS by central GLP-1R agonism could exert antihypertensive effect in the disease condition with excessive sympathoexcitation in SHR. Moreover, administration of liraglutide into 4V, an area with an easy access to AP and NTS, decreased BP transiently. These results suggest that the AP and/or NTS are the possible target sites for antihypertensive effect of liraglutide. Furthermore, the current study is the first to demonstrate that the sites of c-Fos expression by LV liraglutide injection partly change after repeated LV injection of liraglutide for 14 days.

In the present study, both before and after repeated LV injection of liraglutide, LV injection of liraglutide preferentially activated NTS DBH neurons. This result is in agreement with previous report that LV injection of GLP-1R agonist, exendin-4 increased expression of DBH in the NTS^32^. Moreover, we found that the lesion of NTS DBH neurons by treating with anti-DBH-SAP attenuated both the acute depressor effect of the 4V injected liraglutide and chronic antihypertensive effect of the repeated LV injected liraglutide. These results suggest that liraglutide activates NTS DBH neurons to induce antihypertensive effect. Whether liraglutide directly activates NTS DBH neurons remains to be determined. It was reported that fluorescence-labeled liraglutide administered peripherally could reach the brain through blood-brain barrier and its binding was observed in the AP but not NTS^21^. This finding suggests that peripheral liraglutide may not directly interact with the NTS. Our and previous findings collectively indicate that icv liraglutide may directly act on AP and subsequently lead to activation of NTS DBH neurons accompanied by antihypertensive effect. Further investigations are required to definitively elucidate the central neural pathway for the antihypertensive GLP-1 effect.

In previous reports, acute administration of GLP-1 and GLP-1R agonist transiently increase BP in normotensive and hypertensive subjects^4,9,31^. The possible underlying mechanisms involve central cholinergic system^33^, neurohypophysial hormones^33,34^ and medullary catecholamine neurons^31^. In contrast, long-term treatment with GLP-1R agonist decreases BP in hypertensive subjects^4,8–10^. Thus, acute and long-term effects of GLP-1R agonist are opposite in literatures, while underlying mechanism is unclear. In the present study, according to the c-Fos experiments, the initial LV injection of liraglutide activated the hypothalamic PVN and ARC as well as the brainstem AP and NTS, whereas the LV injection of liraglutide after treatment with liraglutide for 14 days failed to activate the hypothalamic PVN and ARC while continuously activating the AP and NTS. Thus, acute action of GLP-1R agonist to transiently increase BP is apparently paralleled with the initial activation of PVN and ARC. The activation of the PVN parvocellular neurons projecting the intermediolateral cell column directly or via RVLM increases sympathetic nerve activity^35,36^. In addition, some neuropeptides in the PVN such as corticotropin-releasing hormone, nesfatin-1 and vasopressin have pressor effect^37,38^. Recently, it was reported that PVN specific GLP-1R deficiency attenuated stress-induced ACTH and corticosterone release and cardiovascular responses, associated with decreased sympathetic drive to the heart^39^. The activation of the melanocortin systems in the ARC also increases sympathetic nerve activity and influences BP^40–43^. Thus, it is plausible that the transient increase in BP by acutely administered GLP-1 and GLP-1R agonist is due to the activation of hypothalamic PVN and ARC. This hypothesis is supported by one previous report that GLP-1 injected into 3V, an area with easy access to ARC and PVN, increases BP^44^.

We have shown that the sustained GLP-1R activation in the CNS decreased the sympathetic nerve activity and counteracted hypertension, in which the activation of the AP and NTS DBH neurons could be implicated. Recently another group reported that GLP-1 hyperpolarized the bulbospinal RVLM neurons using whole cell patch-clamp technique and all the GLP-1-hyperpolarized RVLM neurons showed immunoreactivity for GLP-1R^45^. This study suggests that GLP-1 may directly act on RVLM neurons to decrease the sympathetic nerve activity and reduce BP. This report and our current study taken together, the central mechanisms for antihypertensive effect of GLP-1R agonist may include central GLP-1 action via the AP and NTS pathway and directly on the RVLM. These results by us and others suggests that the activation of GLP-1R in the brainstem is crucial for the antihypertensive effect of central GLP-1.

In the current study, LV liraglutide did not affect SBP in early phase of treatment but decreased SBP at 15 days of treatment. The underlying mechanism is unclear but may include the different effects of GLP-1R agonist on the hypothalamus versus brainstem. We showed acute depressor effect of 4V liraglutide mediated by NTS DBH neurons and acute activation of NTS DBH neurons after LV injection of liraglutide. In acute phase of treatment, LV liraglutide also activated the hypothalamic PVN and ARC in addition to NTS DBH neurons. The pressor effects induced by activation of PVN and ARC could counterbalance the depressor effect induced by activation of NTS DBH neurons. This hypothesis is consistent with the observation at 15 days of liraglutide treatment that LV liraglutide decreased SBP in SHR in parallel with the markedly diminished activation of the hypothalamic PVN and ARC and sustained activation of the NTS DBH neurons.

In the current study, the expression of endothelial nitric oxide synthase (eNOS) mRNA in the kidney cortex of SHR was increased after repeated icv injection of liraglutide for 14 days (Fig. S1A). This result suggests that the eNOS-mediated attenuation of peripheral vascular resistance may underly the antihypertensive effect of central liraglutide. It has been reported that several reagents exert antihypertensive effect via activation of eNOS in SHR^46–49^. There could be an interaction between the eNOS expression and sympathetic nervous system through inflammatory immune system. Activation of the sympathetic nervous system innervating the bone marrow, spleen and peripheral lymphatic systems increases inflammatory cytokines and decreases endothelial progenitor cells, thereby causing vascular endothelial dysfunction to induce hypertension^50–53^. These inflammatory cytokines and endothelial progenitor cells are the factors that regulate the eNOS expression^54^. Therefore, activation of sympathetic nervous systems could decrease the expression of eNOS and exaggerate the hypertension. However, opposite results have also been reported. The limb ischemia-activated sympathetic nervous system increases endothelial progenitor cells in bone marrow via eNOS activation^55,56^. N(omega)-nitro-L-arginine methyl eater (L-NAME), a nonselective NOS inhibitor, reduces renal norepinephrine release induced by renal nerve stimulation in wild type mice but not eNOS knock out mice^57^. These studies suggest that the role of sympathetic nervous system in regulating eNOS expressions might vary depending on specific disease conditions and on acute vs. chronic nature of stimulus. On the other hand, there seems to be a consensus that neuronal NOS (nNOS) has a sympathoinhibitory effect by acting on different sites of the nervous system including nerves in the kidney^58,59^. In current study nNOS mRNA in the kidney cortex of SHR was not changed by treatment with liraglutide (Fig. S1B), suggesting that sympathoinhibitory effect of LV liraglutide is not likely due to interaction with nNOS expression in the kidney. Further investigations are needed to clarify the contribution of NOS expressions in the kidney to the antihypertensive and sympathoinhibitory effects mediated by the liraglutide-activated NTS DBH neurons.

It has been shown that administration of GLP-1 or GLP-1R agonist reduces food intake and body weight (BW)^60–63^. Several clinical studies in obesity patients have reported the association between weight loss and reduction of BP. In some clinical trial in type 2 diabetic patients with overweight or obesity (body mass index ≥25 kg/m^2^), 1-year weight loss of 10% or more was associated with a significant reduction of SBP, around 5 mmHg through 4 years^64^. In an analysis of eight clinical trials in type 2 diabetic patients with obesity (body mass index at baseline: 31.5 ± 5.6 kg/m^2^), the treatment with GLP-1R agonist, exenatide decreased SBP of −5.8 ± 0.7 mmHg in the patients with greater weight loss of −7.0 ± 0.2 kg from baseline, while decreased SBP of −0.5 ± 0.6 mmHg in the patients with smaller weight loss or gain of +1.5 ± 0.1 kg^65^. In our study, weight gain was smaller with liraglutide than saline at 7 days (∆BW + 8.92 g in liraglutide group vs. +16.74 g in control group, p < 0.05) and at 14 days (∆BW + 21.51 g in liraglutide group vs. +29.87 g in control group, p < 0.05). Hence, it is possible that lesser weight gain partly contributed to the antihypertensive effect of repeated icv injection of liraglutide. To assess this possibility, we performed a pair feeding study in which food intake in control saline-injected SHR was restricted to the level in liraglutide-injected SHR. SBP and urinary norepinephrine excretion in the pair-fed saline-injected SHR significantly increased during 14 days, and repeated LV liraglutide injection counteracted the development of hypertension (Fig. 1C) and hyper-excretion of urinary norepinephrine at 14 days (Fig. 2B) to the extents comparable to those in SHR without pair feeding (Figs 1A and 2A). However, we couldn’t exclude possible influence of the BW, because weight gain was still smaller with liraglutide than saline with pair feeding at 14 days (∆BW + 33.58 g in liraglutide group vs. +38.82 g in pair feeding vehicle group, p < 0.05). Nevertheless, the contribution of lesser weight gain to antihypertensive effect of liraglutide may be relatively small, since BW was increasing although weight gain was slower with liraglutide. Furthermore, the magnitude of counteraction against increase in SBP by −39 mmHg (179 ± 2 mmHg vs. 218 ± 13 mmHg) appears to be much greater than that expected from the attenuated weight gain, in view of the quantitative association between weight loss and BP reduction in previous reports^64,65^.

In the current study, repeated LV injection of liraglutide for 15 days did not affect the HR measured 4 hours after LV injection of liraglutide. It was reported that HR was increased by 20 bpm 30 min after LV injection of GLP-1 and by 60 bpm 2 hours after LV injection of exendin-4^31,33^. Another study reported that HR was increased 1 and 2 hours after IP injection of liraglutide, and this response disappeared after 3 hours^16^. Moreover, recent study revealed that the chronotropic effect of IP injection of liraglutide was diminished in mice with cardiomyocyte specific deficiency of GLP-1R, and GLP-1R in the heart interacted with autonomic nervous system to regulate HR^66^. These reports investigated acute effect of initial GLP-1 or GLP-1R agonist injection on HR. Hence, the different results between our and previous studies could reflect the different time points investigated and/or the difference between repeated and initial injection of GLP-1 or GLP-1R agonist. Furthermore, in our study 4V injection of liraglutide did not affect HR in the time scale of minutes in anesthetized rats, whereas it was reported that 4V injection of exendin-4 increased HR for several hours with peak at 6.5 hours after administration in non-anesthetized free moving rats^67^. These different experimental conditions may have caused different results.

There are several limitations in current study. First, we did not perform direct recording of sympathetic nerve activity in both acute and chronic treatment with liraglutide. Whether acute depressor effect of 4V injection of liraglutide is accompanied with decreased sympathetic nerve activity has not been determined yet. We assessed urinary norepinephrine excretion as a marker for systemic sympathetic nerve activity under chronic treatment with liraglutide. Previous reports suggest a tight correlation between urinary norepinephrine level and sympathetic nerve activity recorded in muscle, kidney and lumber in several disease conditions^68–71^. Based on this relationship, urinary norepinephrine has been widely used as a reliable marker for sympathetic nerve activity and hence also used in current study. Second, we measured BP indirectly by tail-cuff method instead of telemetry method that has been shown to accurately monitor blood pressure in long-term studies^72,73^. Tail-cuff method-induced restrained stress affecting BP cannot be eliminated completely. However, in current study tail-cuff method without heating was used to avoid thermal stress and rats were habituated to the procedure for 7 days before experiment to minimize the circumstantial stress. There are several reports showing that BP measured by tail-cuff method is strongly correlated with that by telemetry method and with that by simultaneous arterial catheter method in rodents including SHR used in current study^74–76^. Hence, we considered that assessment of chronic effect of liraglutide on BP in SHR in current study is acceptable.

In conclusion, we have demonstrated that long-term central treatment with GLP-1R agonist attenuates the development of hypertension via mechanisms involving the activation of brainstem DBH neurons and suppression of sympathetic nerve activity in SHR. Both peripheral and central injection of liraglutide increased c-Fos expression in the same area of the brain in SHR. These results suggest that the activation of specific brain areas by central GLP-1R agonist demonstrated in current study may underlie the effects reported in the recent clinical studies that treatment with GLP-1R agonists significantly reduced BP and cardiovascular events in type-2 diabetic patients^1–3^. Furthermore, we showed the antihypertensive effect of GLP-1R agonist in addition to and independently of well-established anti-diabetic and anti-obese effects, suggesting that GLP-1R agonist is beneficial especially to the hypertensive patients with diabetes and/or obesity.

## Methods

### Animals

Male SHR and control WKY rats aged 6 weeks (Hoshino Laboratory Animals, Ibaraki, Japan) were maintained on a 12 h light/dark cycle (19:30 light off) and given conventional food (CE-2; Clea, Osaka, Japan) and water ad libitum. All animal procedures were conducted in compliance with protocols approved by Jichi Medical University Animal Care and Use Committee, and all experiments were carried out in accordance with the approved protocols.

### LV cannulation and BP measurement

Rats were anesthetized by IP injection of Avertin (tribromoethanol, 200 mg/kg, ip). A stainless steel guide cannula (26-gauge) was stereotaxically inserted into LV (0.8 mm caudal to the bregma, 1.5 mm lateral from midline and 3.2 mm below the surface of the skull). The injector needle extended 0.6 mm below the guide cannula. Rats were allowed to recover from the operation for 10 days while they were habituated to handling. Liraglutide (Novo Nordisk Pharma Ltd., Tokyo, Japan) 0.9 µg/3 µl dissolved in vehicle (saline; 0.9% NaCl) or 0.9% NaCl 3 µl (vehicle) was injected once a day for 15days. SBP and HR were measured using a tail-cuff system (Model MK-2000, Muromachi Co. Ltd., Tokyo, Japan) in conscious rats 4 hours after LV injection of liraglutide or vehicle, and the measurements were performed every three days up to 15 days in SHR and at 1 and 2 weeks in WKY rats. Rats were allowed to habituate to the procedure for 7 days before experiments were performed. SBP and HR values were averaged from the three consecutive recordings obtained from each rat. At the end of the experiments, sections of the forebrain were histologically examined to verify the position of the cannulas. The rats in which cannulas were outside LV were excluded from the data analysis. For pair-feeding, the amount of food consumed by the liraglutide-treated group over the course of 24 h was measured at 9:00, and a corresponding amount of pellets was given to the pair-fed group over a 24-h period. The food intake over 24 h was calculated by weighing the remaining food pellets every day. SBP and HR were measured as mentioned above in the pair-fed SHR at 1 and 2 weeks.

### NTS microinjection, 4V cannulation and BP measurement

A separate group of rats were used to assess the consequences of lesions of NTS DBH neurons on changes in blood pressure by 4V injection of liraglutide. Rats were anesthetized with avertin (200 mg/kg, IP). Glass micropipettes filled with blank-SAP (Advanced Targeting Systems, San Diego, CA) 6 ng/200 nl or anti-DBH-SAP (Advanced Targeting Systems, San Diego, CA) 40 ng/200 nl dissolved in saline were stereotaxically placed into the NTS (12.5 mm caudal to the bregma, 0.5 mm lateral from midline and 8.9 mm below the surface of the skull). Microinjections were made bilaterally. A total volume (200 nl) of injection was made over 2 min after which the pipette was left in place for additional 10 min to prevent reflux of fluid from the pipette track. For 4V injection, the cannula was placed stereotaxically into the 4V (11.5 mm caudal to the bregma, in the midline and 8.6 mm below the surface of the skull) and the position of cannula was histologically verified at the end of the experiment as described above. Two weeks after NTS microinjection of saporin, 4V cannulated rats were anesthetized with urethane (1.0 g/kg, IP). The left femoral artery was cannulated, and the arterial line was connected to a pressure transducer (Amplifier Case 7903, Sanei Cardio Co. Ltd., Tokyo, Japan) for data recording and analysis (LabScribe2, Worx Systems Inc., Dover, NH) of BP and HR. Then rats were placed in a stereotaxic apparatus in a prone position, and BP and HR were monitored after 4V injection of liraglutide (0.9 µg/3 µl).

### Immunohistochemistry for c-Fos and DBH

Immunohistochemistry for c-Fos and DBH were performed after LV injection of liraglutide (0.9 µg/3 µl) and IP injection of liraglutide (90 µg/kg). Four hours after injection of liraglutide or saline, rats were anesthetized with isoflurane and perfused transcardially saline containing heparin (20 U/ml) for 3 min and then 4% paraformaldehyde in 0.1 M phosphate buffer (PB) containing 0.2% picric acid for 20 min. The brains were removed and postfixed in the same fixative for 1 h and in 0.1 M PB containing 15% sucrose overnight at 4 °C. They were then transferred to 30% sucrose solution in 0.1 M PB for 2 days. The brains were embedded in Tissue-Tek OCT compound (Sakura Finetechnical Co. Ltd., Tokyo, Japan) and frozen on dry ice and kept at −80 °C until sectioning. Coronal sections with 40 µm thickness were cut with a freezing microtome and collected at 160 µm intervals. Sections were rinsed in phosphate buffered saline (PBS) and then incubated in 0.3% H_2_O_2_ for 20 min. After rinsing, sections were blocked with 2% bovine serum albumin and 2% normal goat serum for 30 min and incubated with rabbit anti-c-Fos antibody (Ab-5; Calbiochem, San Diego, CA; 1:2000) overnight at 4 °C. Then the sections were rinsed and incubated with biotinylated goat anti-rabbit IgG for 30 min. After rinsing, sections were incubated with avidin-biotin complex (ABC) reagent for 30 min (Vector Laboratories; 1:500). After rinsing in PBS, color was developed with a nickel-diaminobenzidine (DAB) solution (0.3% nickel ammonium sulfate, 0.02% DAB, and 0.005% H_2_O_2_ in 0.05 M Tris buffer) for 5 min. For double-labeling immunohistochemistry for c-Fos with DBH, the process of DBH staining was added. After rinsing in PBS, sections were treated with an avidin and biotin blocking solution (Vector Laboratories) and then incubated with mouse anti-DBH antibody (MAB308; Millipore, Billerica, MA, 1:500) diluted in a blocking solution overnight at 4 °C. After rinsing, sections were incubated with biotinylated goat anti-rabbit antibody for 30 min and incubated in ABC reagent for 30 min. Then the sections were rinsed in PBS and Tris buffer, and color was developed with a DAB solution (0.02% DAB and 0.005% H_2_O_2_ in Tris buffer). Slices were then rinsed, mounted on slides, and coverslipped with Entellan new (Merck, Darmstadt, Germany).

The number of c-Fos-IR and DBH-IR cells per section were counted in PVN, supraoptic nucleus (SON) and ARC between −1.3 and −3.3 mm or in NTS, RVLM and caudal ventrolateral medulla (CVLM) between −12.3 and −14.3 mm from bregma. For double immunostaining, c-Fos-IR cells, DBH-IR cells, and dually IR cells were counted in the sections containing NTS between −13.3 and −14.3 mm from bregma. Cell counting was conducted using Find Maxima function in ImageJ software (NIH), which was validated by manual counting of IR cells. Three to five sections were averaged for each rat. The fraction of c-Fos-IR cells in DBH-IR cells, expressed as percentage, was averaged for all sections.

### Urinary norepinephrine excretion measurement

Urinary norepinephrine excretion was measured as an index of overall sympathetic activity at 2 weeks of repeated administration of liraglutide or vehicle. Urine samples were collected with the metabolic cages KN-649 (Natsume Co. Ltd., Tokyo, Japan) for 24 h using the 50-ml collecting tubes contained 6N HCl to prevent the autooxidation of catecholamines. The urinary norepinephrine concentrations were measured with high-performance liquid chromatography (SRL Inc., Tokyo, Japan).

### Real-time PCR to determine mRNA expression of eNOS and nNOS in the kidney

Kidney cortex was excised from the left and right sides under anesthesia with isoflurane. Total RNA was isolated using TRIzol (Invitrogen, Madison, WI) and treated with RQ1-DNase (Promega, Madison, WI). First-strand cDNA synthesis was completed using ReverTra Ace kit (Toyobo, Osaka, Japan). Quantitative RT-PCR assay was performed using SYBR Premix Ex Taq II polymerase in Thermal Cycler Dice (Takara Bio, Tokyo, Japan). Expression levels of mRNAs were calculated by the ΔΔCT method of relative quantification, and corrected by 18 S Ribosomal RNA. The base sequences of primers used are as follows.

Rat eNOS:                      forward;   5′-AGCTGGATGAAGCCGGTGAC-3′

reverse; 5′-CCTCGTGGTAGCGTTGCTGA-3

Rat nNOS:                     forward;  5′-CCTATGCCAAGACCCTGTGTGA-3′

reverse; 5′-CATTGCCAAAGGTGCTGGTG-3′

18S ribosomal RNA: forward; 5′-TTCGAACGTCTGCCCTATCAA-3′

reverse; 5′-ATGGTAGGCACGGCGACTA-3′.

### Statistical analysis

Data are expressed as means ± S.E. Scatter plot was included in several bar graphs. Data were analyzed by unpaired or paired Student’s t test or by one-way or repeated measures two-way ANOVA with treatment (saline vs. liraglutide) and time as factors. Post hoc multiple comparisons were made using Turkey’s post hoc test. P < 0.05 was considered significant.

## Supplementary information

Supplementary Information
