# Supplementary material for: Central Glucagon-like Peptide-1 Receptor Signaling via Brainstem Catecholamine Neurons Counteracts Hypertension in Spontaneously Hypertensive Rats
Source: Sci Rep. 2019 Sep 19;9:12986. doi: 10.1038/s41598-019-49364-x (PMC6753091; doi:10.1038/s41598-019-49364-x)
Supplement: Supplementary file 1 — Supplementary Information [file 41598_2019_49364_MOESM1_ESM.pdf]

Supplementary information

Central Glucagon-like Peptide-1 Receptor Signaling via Brainstem Catecholamine Neurons Counteracts Hypertension in Spontaneously Hypertensive Rats

Kenichi Katsurada[1,2,5], Masanori Nakata[1,3], Toshinobu Saito[1,2], Boyang Zhang[1], Yuko Maejima[4], Shyam S. Nandi[5], Neeru M. Sharma[5], Kaushik P. Patel[5], Kazuomi Kario[2], Toshihiko Yada[1,6,7] *

[1]Division of Integrative Physiology, Department of Physiology

[2]Division of Cardiovascular Medicine, Department of Internal Medicine

Jichi Medical University School of Medicine, Shimotsuke, Tochigi 329-0498, Japan

[3]Department of Physiology, Wakayama Medical University School of Medicine, Wakayama 641-8509, Japan

[4]Department of Pharmacology, Fukushima Medical University School of Medicine, Fukushima 960-1295, Japan

[5]Department of Cellular and Integrative Physiology, University of Nebraska Medical Center, Omaha, NE 68198-5850

[6]Center for Integrative Physiology, Kansai Electric Power Medical Research Institute, 1-5-6 Minatojimaminamimachi, Chuou-ku, Kobe 650-0047, Japan

[7]Division of System Neuroscience, Kobe University Graduate School of Medicine, Kobe 650-0017, Japan

*Correspondence: tyada@jichi.ac.jp

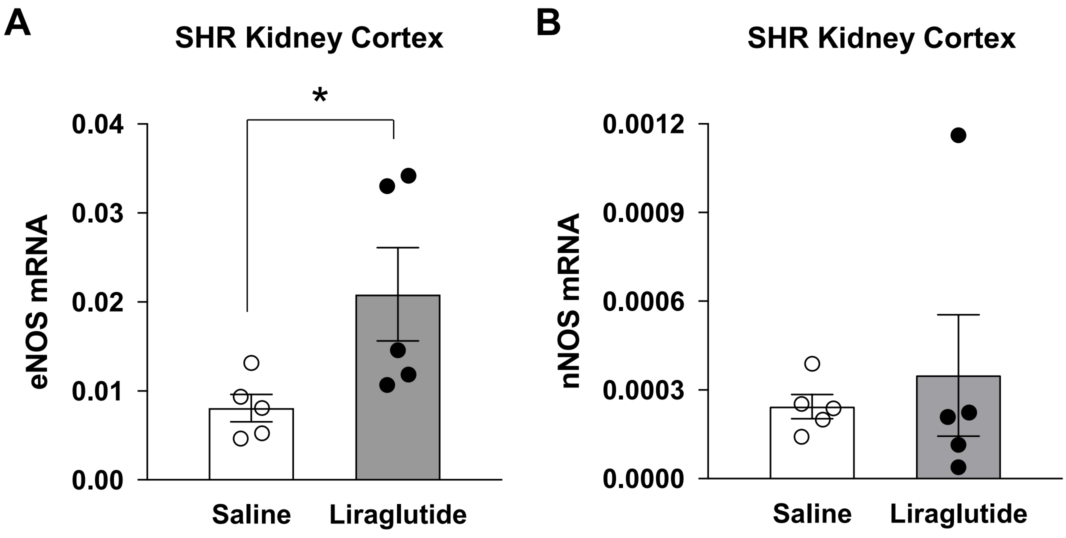

Supplementary Figure S1. Repeated LV injection of liraglutide increases eNOS but not nNOS mRNA expression in kidney cortex of SHR

eNOS (A) and nNOS (B) mRNA expression in the kidney cortex of SHR after repeated LV injection of saline or liraglutide for 2 weeks (n = 5). Each bar represents mean ± SE. $^*P < 0.05$ between liraglutide and saline.
